# Aurora Kinase A Inhibition Potentiates Platinum and Radiation Cytotoxicity in Non-Small-Cell Lung Cancer Cells and Induces Expression of Alternative Immune Checkpoints

**DOI:** 10.3390/cancers16162805

**Published:** 2024-08-09

**Authors:** Huijie Liu, Ayse Ece Cali Daylan, Jihua Yang, Ankit Tanwar, Alain Borczuk, Dongwei Zhang, Vincent Chau, Shenduo Li, Xuan Ge, Balazs Halmos, Xingxing Zang, Haiying Cheng

**Affiliations:** 1 Department of Oncology, Montefiore Medical Center/Albert Einstein College of Medicine, Bronx, NY 10461, USA; hliu@fulgentgenetics.com (H.L.); jihua.yang@einsteinmed.edu (J.Y.); ankit.tanwar@einsteinmed.edu (A.T.);; 2 Department of Pathology, Northwell Health, Staten Island, NY 10305, USA; 3 Department of Pathology and Laboratory Medicine, Indiana University, Indianapolis, IN 15705, USA; dz11@iu.edu; 4 Department of Medicine, Mayo Clinic Comprehensive Cancer Center, Jacksonville, FL 32224, USA; li.shenduo@mayo.edu; 5 Department of Hematology/Oncology, Kaiser Permanente, Modesto, CA 95356, USA; 6 Department of Microbiology and Immunology, Albert Einstein College of Medicine, Bronx, NY 10461, USA

**Keywords:** aurora kinase A, immune checkpoints, platinum, NSCLC, radiation

## Abstract

**Simple Summary:**

Despite major advances in treating non-small-cell lung cancer (NSCLC), the five-year survival rates for patients without specific genetic mutations remain low. This study aims to find better ways to treat these patients. We focused on a protein called Aurora kinase A (AURKA), which our prior study suggested may play a role in modulating resistance to the common cancer drug cisplatin. We looked at how AURKA affects the sensitivity of NSCLC cells to platinum-based chemotherapy and radiation. In lab tests, AURKA levels increased after cisplatin treatment. Blocking AURKA with a drug called alisertib or using a genetic method made cisplatin and radiation more effective, causing tumors to shrink in mouse models. The treated cancer cells showed more DNA damage and underwent cell death. Blocking AURKA also increased markers that help the immune system recognize cancer. Our study suggests that combining AURKA inhibitors with other treatments could improve outcomes for NSCLC patients.

**Abstract:**

Despite major advances in non-small-cell lung cancer (NSCLC) treatment, the five-year survival rates for patients with non-oncogene-driven tumors remain low, necessitating combinatory approaches to improve outcomes. Our prior high-throughput RNAi screening identified Aurora kinase A (AURKA) as a potential key player in cisplatin resistance. In this study, we investigated AURKA’s role in platinum and radiation sensitivity in multiple NSCLC cell lines and xenograft mouse models, as well as its effect on immune checkpoints, including PD-L1, B7x, B7-H3, and HHLA2. Of 94 NSCLC patient tumor specimens, 91.5% tested positive for AURKA expression, with 34% showing moderate-to-high levels. AURKA expression was upregulated following cisplatin treatment in NSCLC cell lines PC9 and A549. Both AURKA inhibition by alisertib and inducible AURKA knockdown potentiated the cytotoxic effects of cisplatin and radiation, leading to tumor regression in doxycycline-inducible xenograft mice. Co-treated cells exhibited increased DNA double-strand breaks, apoptosis, and senescence. Additionally, AURKA inhibition alone by alisertib increased PD-L1 and B7-H3 expression. In conclusion, our study demonstrates that AURKA inhibition enhances the efficacy of platinum-based chemotherapy in NSCLC cells and modulates the expression of multiple immune checkpoints. Therefore, combinatory regimens with AURKA inhibitors should be strategically designed and further studied within the evolving landscape of chemo-immunotherapy.

## 1. Introduction

Although there have been significant advancements in non-small-cell lung cancer (NSCLC) therapies, NSCLC remains the leading cause of cancer-related deaths in the U.S. [1]. One of the breakthroughs in NSCLC treatment has been the introduction of immune checkpoint inhibitors over the last decade. The incorporation of immunotherapy in stage III NSCLC increased median overall survival from 29 months to 47.5 months [2]. In the non-oncogene-driven metastatic NSCLC, the addition of immunotherapy nearly doubled the median overall survival. However, despite these improvements, five-year median survival rates remain disappointingly low at around 20–25% for metastatic NSCLC and 43% for stage III NSCLC [2,3]. Hence, strategies to increase the efficacy of the commonly used NSCLC treatment modalities are essential. Exploring targeted agents in a combinatory fashion with the current treatment agents in NSCLC is a plausible strategy to improve clinical outcomes. Moreover, understanding the interplay between these targeted agents and immune checkpoints is crucial in the current treatment landscape of NSCLC.

We previously conducted a high-throughput RNAi screening platform to explore candidate genes playing a role in cisplatin resistance [4]. We selected the most depleted shRNAs in cisplatin-treated cells based on the hypothesis that these target genes are vital in cell survival under cisplatin treatment. The level of shRNA targeting Aurora kinase A (AURKA) was seriously depleted (−113-fold change) in cisplatin-treated PC9 cells, indicating an important role of AURKA in cisplatin resistance [4].

AURKA is a member of the Aurora family of serine/threonine kinases [5]. It regulates the cell cycle by controlling centrosome maturation, G2/M transition, mitosis entry, and cytokinesis [6,7,8]. Gene amplification and overexpression of AURKA have been detected in many cancers, including lung, ovarian, pancreatic, breast, and bladder cancer [9,10,11,12]. Overexpression of AURKA has been reported to play a role in various cellular processes that contribute to carcinogenesis and tumor progression [13,14,15,16,17,18,19,20,21]. It has also been shown to correlate with poor patient survival outcomes in NSCLC patients [22,23].

AURKA has been implicated in cancer-directed therapy resistance in multiple studies [13,23,24,25]. Lung cancer cell lines resistant to cisplatin were reported to have a higher expression of AURKA and AURKA inhibition; in addition, cisplatin led to increased cytotoxicity in NSCLC cell lines [23]. MLN8237 (alisertib), an AURKA inhibitor, was reported to bind and inhibit the kinase activity of AURKA [26]. In vivo studies have also suggested that AURKA is a plausible cancer therapeutic target. For instance, gastric cancer xenografts with cisplatin resistance showed tumor shrinkage when treated with MLN8237 [27]. In vivo NSCLC xenograft treated with the combination of MLN8237 and radiation achieved superior tumor inhibition [22].

Given these promising preclinical data on MLN8237, multiple clinical trials have been conducted with MLN8237. However, the overall single-agent efficacy of MLN8237 in clinical trials has been underwhelming. The reasons why MLN8237 has underperformed in clinical trials compared to the preclinical data have garnered attention. Recently, it has been proposed that Aurora A inhibition may alter the tumor immune microenvironment, compromising the anti-tumor efficacy of MLN8237 [28]. Investigation of interactions between tumor cells and the surrounding immune microenvironment is critical in the current NSCLC treatment landscape. The introduction of immunotherapy led to unprecedented improvement in the median survival of NSCLC patients. However, this durable survival benefit remains limited to a small subset of patients. The immunotherapy resistance mechanisms are multifactorial and rely on intrinsic cellular changes and dynamic interactions of cancer cells with the surrounding immune and stromal cells [29]. The commonly used immune checkpoint inhibitors in NSCLC treatment target the PD-1/PD-L1 pathway. However, many NSCLC patients lack PD-L1 yet express other immune checkpoints. Alternative immune evasion pathways using group III molecules of the B7-CD28 immune checkpoint family, which are B7x (B7-H4/B7S1), B7-H3 (CD276), and HHLA2 (B7H7/B7-H5/B7y), are expressed in 60–90% of NSCLC patients [30,31,32]. Targeting these immune evasion pathways may prove effective, especially in tumors resistant to PD-1/PD-L1 pathway inhibitors or lacking PD-L1 expression. Combination strategies are explored to decrease the risk of resistance and expand immunotherapy benefits to a larger group of patients [29]. AURKA inhibition may induce immune checkpoint expression in cancer cells [28] and alter the composition or intrinsic cellular characteristics of cancer, immune, or stromal cells. Hence, rationally designed combination therapies incorporating immune checkpoint inhibitors and AURKA inhibition may be a plausible strategy.

As summarized above, Aurora kinase A inhibition as a cancer therapeutic target is an ongoing area of research. Its integration into NSCLC regimens needs further refinement, as the lung cancer treatment landscape has changed significantly with the advent of immunotherapy. To assess whether combining AURKA inhibition with cisplatin or radiation increases its efficacy, we performed in vitro and in vivo studies investigating cell viability and tumor size. To explore potential immune evasion pathways induced by the use of AURKA inhibitors, we examined the effect of AURKA inhibition on the expression of immune checkpoints PD-L1, B7x, B7-H3, and HHLA2. This study presents preclinical data to support combination regimens of AURKA inhibition in NSCLC treatment, details changes in cellular processes under these treatment combinations, and describes the effect of AURKA inhibition on various immune checkpoints.

## 2. Materials and Methods

Cell lines and material: The NSCLC cell lines A549, H1703, and H460 were obtained from the American Type Tissue Collection (Manassas, VA, USA). PC9 cells were a gift from Dr. Susumu Kobayashi, Harvard Medical School, Boston, MA, USA. Lung cancer cells were grown in RPMI 1640 supplemented with 10% FBS and 1× Antibiotic/Antimycotic (Catalog no. 15240096, Invitrogen, Carlsbad, CA, USA). MLN8237 was obtained from Selleck Chemicals (Catalog no. S1133, Houston, TX, USA), and cisplatin was obtained from Sigma-Aldrich (Catalog no. 1134357, St. Louis, MO, USA).

High-throughput RNAi screening: A high-throughput RNAi screening platform was performed as previously described [4]. In brief, PC9 cells infected by the shRNA library were treated with cisplatin and control DMSO vehicle. Compared to DMSO-treated cells, the most depleted shRNAs in cisplatin-treated surviving cells were reviewed as candidate genes essential for cell survival under cisplatin treatment.

Immunoblotting and antibodies: 10% SDS-PAGE gel was used to separate proteins which were transferred onto nitrocellulose membrane by Bio-Rad as described previously [4]. For Western blot analysis, antibodies against AURKA (catalog no. 91590), phospho-Aurora A (Thr288) (catalog no. 3079), total PARP (catalog no. 9532), cleaved PARP (Asp214) (catalog no. 5625), and glyceraldehyde-3-phosphate dehydrogenase (GAPDH) (catalog no. 5174) were sourced from Cell Signaling Technology (Boston, MA, USA). Antibody against phospho-Histone H2AX (Ser 139) (SKU 05-636-25UG) was purchased from Millipore (Temecula, CA, USA).

Immunohistochemistry: As previously described [33,34], formalin-fixed primary lung tumor tissue sections were deparaffinized and subjected to antigen retrieval treatment with sodium citrate (10 mM, pH 6.0) after rehydration. Endogenous peroxidase activity was blocked by Dako peroxidase blocking reagent (Dako Corporation, Carpinteria, CA, USA). AURKA antibody, purchased from GeneTex (catalog no. GTX13824), was used at a dilution of 1:100 at room temperature. Subsequently, DakoCytomation LSAB2 system-HRP (Dako Corporation, Carpinteria, CA, USA) was utilized. Biotinylated link universal and streptavidin-HRP were added, followed by DAB chromogen (Dako Corporation, Carpinteria, CA, USA) and hematoxylin nuclear counterstaining. Two independent investigators assessed the expression of AURKA using immunohistochemistry (IHC) in formalin-fixed paraffin-embedded tissue specimens. The semiquantitative immunohistochemical evaluation was conducted using the H-score. Samples with positive AURKA staining, whether in the cytoplasm, nucleus, or both, were considered positive cases. The H-score was determined by multiplying the percentage of staining cells (proportion score) by an ordinal value corresponding to the maximum intensity level in the specimen (0 = none, 1 = weak, 2 = moderate, 3 = strong).

Drug combination studies with Clonogenic survival assay: As previously described [35], cells in the logarithmic growth phase were seeded in triplicates in 6-well tissue culture plates with 200 cells per well in media alone and 500 cells per well in drug-supplemented media. Drug doses are documented in the figures, and the radiation dose was 2Gy in all experiments. After 7–14 days, colonies were fixed with 70% ethanol and stained with 0.5% crystal violet. Colonies with more than 50 cells were considered surviving colonies. Survival was quantified as the relative plating efficiency as compared to control cells. The Bliss Model was used to assess the effects of drug combinations [36]. f1, f2 and f12 represent the effects of single drug 1, single drug 2, and the combination of drugs 1 and 2, respectively. The Bliss combination index is defined as (CI Bliss) = (f1 + f2 − f1 × f2)/f12. The drug–drug interactions (or drug–AURKA knockdown interaction) were classified as synergism if CI < 1, antagonism if CI > 1, and additive if CI = 1. CIs were also calculated by Calcusyn software V2 (Biosoft, Cambridge, UK) based on the method of Chou and Talalay [37]. Similar CI trends were observed with both methods. In the figures, Δ is used to denote a synergistic effect of combined treatments.

MTS cell growth assay: Cells were plated at a density of 3000 cells per well, or 1500 cells for siRNA experiments, in 96-well plates with RPMI 1640 medium supplemented with 10% FBS on day 0. On day 1, they were treated with increasing doses of cisplatin, doxycycline, or AURKA inhibitor (doses as indicated in the figures). Radiation, if used, was given at a dose of 2 Gy. After incubation with the treatments for three days, cell viability was assessed on day 4 using the MTS assay kit, following the protocol provided by Promega, Madison, WI, USA. Each experimental condition was replicated in three wells.

Small interfering RNA (siRNA) knockdown: Knockdown of AURKA was achieved using siRNA pools targeting AURKA (ON-TARGET plus SMARTpool) obtained from GE Dharmacon RNAi Technologies (Lafayette, CO, USA). Nontargeting siRNA Pools were used as the negative control. SiRNA was introduced using Lonza Amaxa cell line nucleofector kit C according to the manufacturer’s instructions (Morristown, NJ, USA). The extent of AURKA knockdown at various time points was evaluated by immunoblot analysis using samples from the pools of transfected cells.

Inducible shRNA knockdown: The Inducible Dharmacon TRIPZ Lentiviral shRNA was utilized following the manufacturer’s instructions for the inducible knockdown of AURKA (TRIPZ Human AURKA shRNA, Clone ID: V2THS_12364, GE Dharmacon, CO, USA). Doxycycline induction of knockdown of AURKA was confirmed by Western blotting.

Xenograft in vivo studies: Four- to six-week-old athymic nu/nu mice were purchased from the Jackson laboratories. Each mouse was inoculated with 5 × 106 PC9A8 cells (PC9 cells transfected with AURKA shRNA lentivirus) in 0.2 mL subcutaneously into the left flank of nude mice. On day 9, when the tumor size was larger than 100 mm^2^ but less than 200 mm^2^, drug treatment was initiated. The twenty mice were divided randomly into four groups. The control group received no treatment; one group was treated with cisplatin, 5 mg/kg, twice a week by intraperitoneal (i.p.) injection; one group was treated with doxycycline, 1 mg/mL in drinking water (5% sucrose was used to mask the bitter flavor and the doxycycline was changed every other day); and one group was treated with cisplatin plus doxycycline. The tumor size was measured twice a week. The tumor volume was calculated by the formula: L × W2/2, where L is the tumor length, and W is the tumor width.

Senescence-associated β-galactosidase staining: PC9 and A549 cell lines were treated with MLN8237 and/or cisplatin for 48 h in triplicates. The treated cells were plated into 6-well plates one day prior to staining. Cells were fixed and stained per the manufacturer’s protocol (Senescence β-Galactosidase Staining kit, Cell Signaling #9860, Boston, MA, USA). Stained cells were imaged with a Zeiss Axio Observer inverted scope with differential interference contrast brightfield microscopy (DIC). Ten random images were collected, and cells were counted with ImageJ software version 1.53.

Flow cytometry of cell cycle stages: Flow cytometric analysis of the cell cycle was conducted with propidium iodide DNA staining (Abcam, Cambridge, MA, USA, catalog no. 14082). In brief, adherent cells were harvested and washed in PBS followed by fixing in cold 70% ethanol for 30 min at 4 °C. Prior to flow cytometry analysis, cells were washed twice in PBS and centrifuged. The cell pellet was resuspended in 50 µL of a 100 µg/mL stock of RNase and 200 µL propidium iodide (from a 50 µg/mL stock solution). Cells were incubated at 37 °C for 20–30 min in the dark followed by flow cytometry analysis (Cytek Aurora, Cytek Biosciences, Fremont, CA, USA).

Flow cytometry for immune checkpoints: Flow cytometry was used to detect PD-L1, B7-H3, B7x, and HHLA2 expression on treated A549 and H1703 cells, as previously described in [31]. Cells pretreated with MLN8237 were stained with APC anti-hPD-L1 (clone 29E.2A3), PE/Cy7 anti-hB7-H3 (clone MIH42), PE anti-hB7x (clone MIH43), or isotype controls for 1 h at 4 °C. Fluorophore-conjugated antibodies were purchased from BioLegend. The staining of HHLA2 was performed by first incubating cells with primary mouse anti-hHHLA2 mAb (clone B5B5) or control mouse IgG1 (clone MOPC-21) for 40 min at 4 °C, followed by staining with APC polyclonal goat F(ab′)2 anti-mouse IgG Fc (eBioscience, Frankfurt, Germany) for 30 min at 4 °C. Cells were washed after staining and analyzed on Cytek Aurora (Cytek Biosciences, Fremont, CA, USA). Data were analyzed with FlowJo (FlowJo, LLC, Ashland, OR, USA).

Statistics: All data are presented as means ± SD from at least triplicate experiments. Statistical analysis of cell lines was conducted using one- or two-way ANOVA, as appropriate, with GraphPad Prism version 9 (GraphPad Software, Boston, MA, USA). AURKA expression levels, assessed by IHC, were compared between groups defined by mutation status and histology with the Kruskal–Wallis test. A two-sided *p*-value < 0.05 was considered statistically significant. The following notation was used to symbolize statistical significance in the figures: *p* < 0.05, ** *p* < 0.01, and *** *p* < 0.001.

## 3. Results

### 3.1. AURKA Expression in Human NSCLC Tumor Specimens and Its Change to Cisplatin in NSCLC Cell Lines

**Figure 1:**
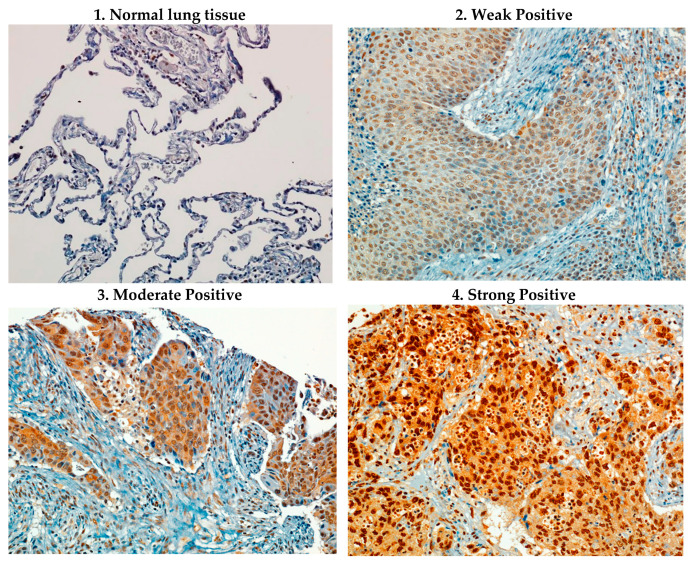
AURKA expression in lung cancer. Representative AURKA IHC staining of tissue microarrays.

To determine the expression patterns of AURKA, immunohistochemistry staining of AURKA on a tissue microarray of 94 well-annotated NSCLC lung cancer tumor samples from patients with stage I-III lung cancer who underwent resection between 2010 and 2012 was pursued (Figure 1).

AURKA was found to be expressed in 91.5% of tumors with 57.5% of tumors having weak expression and 34.0% of tumors having moderate-to-strong expression (Table 1). There was no significant association between AURKA expression and histologic subtype nor the presence of EGFR or KRAS mutations.

**Figure 2:**
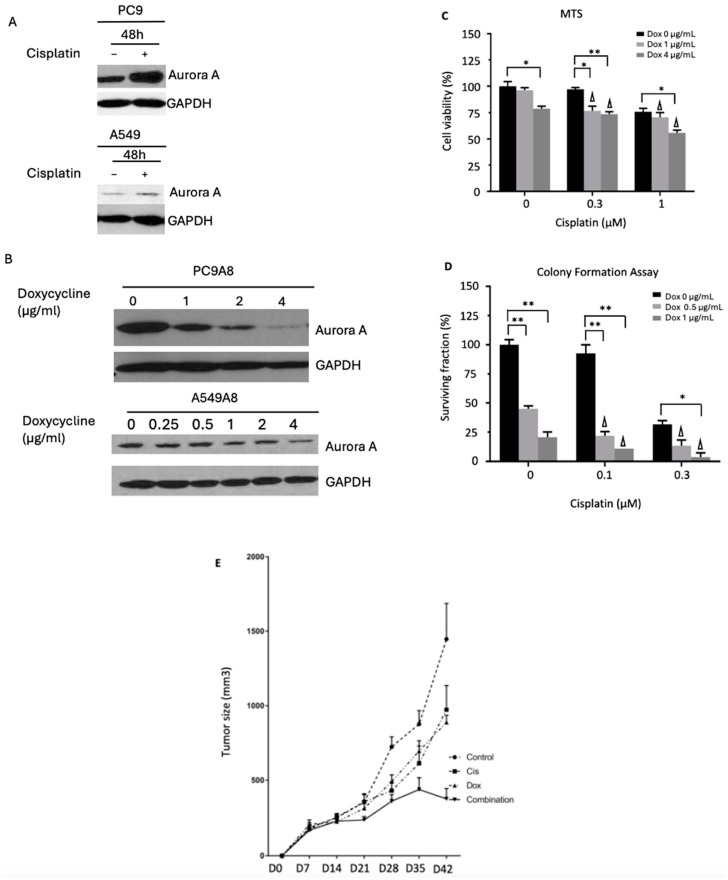
Effect of AURKA knockdown in combination with cisplatin treatment. (**A**) Aurora A protein expression by Western blot increases with cisplatin treatment. (**B**) Aurora A protein expression decreases when doxycycline-inducible cell lines are treated with varying amounts of doxycycline as detected by Western blot. (**C**) Cell viability by MTS assay reveals that doxycycline-inducible Aurora A knockdown leads to decreased cell viability in PC9A8 cell line. Addition of cisplatin to Aurora A knockdown works synergistically to decrease cell viability (results for A549A8 can be found in the Supplementary Figure S1). (**D**) Clonogenic assay reveals that doxycycline-inducible Aurora A knockdown reduces colony formation in the PC9A8 cell line. The addition of cisplatin to Aurora A knockdown works synergistically to decrease tumor cell colony formation (results for A549A8 can be found in the Supplementary Figure S1). (**E**) Xenograft studies reveal that both cisplatin and Aurora A knockdown when used alone slow down tumor growth. When Aurora A knockdown is combined with cisplatin, actual tumor size reduction can be noted. The following notation was used to symbolize statistical significance in the figures: * *p* < 0.05, ** *p* < 0.01. Δ is used to denote a synergistic effect of combined treatments.

To explore this potential target identified in RNAi screening results, we examined whether AURKA expression would be altered upon treatment with cisplatin in NSCLC cell lines. The expression of AURKA was upregulated in NSCLC cell lines PC9 and A549 when treated with cisplatin (Figure 2A).

### 3.2. AURKA Knockdown and MLN8237 (Inhibitor)-Sensitize NSCLC Cells to Cisplatin and Radiation

Next, we examined the effect of AURKA knockdown on cisplatin efficacy. Higher doses of doxycycline concentration in the inducible shRNA knockdown system correlated with lower levels of AURKA expression (Figure 2B). AURKA knockdown alone decreased cell viability and colony formation in PC9A8 and A549A8 cells (Figure 2C,D). In addition, AURKA knockdown improved the sensitivities of PC9 and A549 lung cancer cells to cisplatin in a synergistic manner in cell viability and colony formation assays (CI ˂ 1) (Figure 2C,D).

The effects of AURKA knockdown and its combination with cisplatin on tumor size were also studied in vivo with xenograft studies. The xenograft model of doxycycline-inducible AURKA knockdown NSCLC tumor treated with cisplatin or doxycycline alone demonstrated slower growth of tumor mass compared to that of untreated mice (Figure 2E). When the mice were treated with cisplatin in the setting of AURKA knockdown, an actual reduction in tumor size was observed after day 35 compared to either treatment alone. These results further indicate that AURKA knockdown sensitizes lung cancer to cisplatin (Figure 2E).

**Figure 3:**
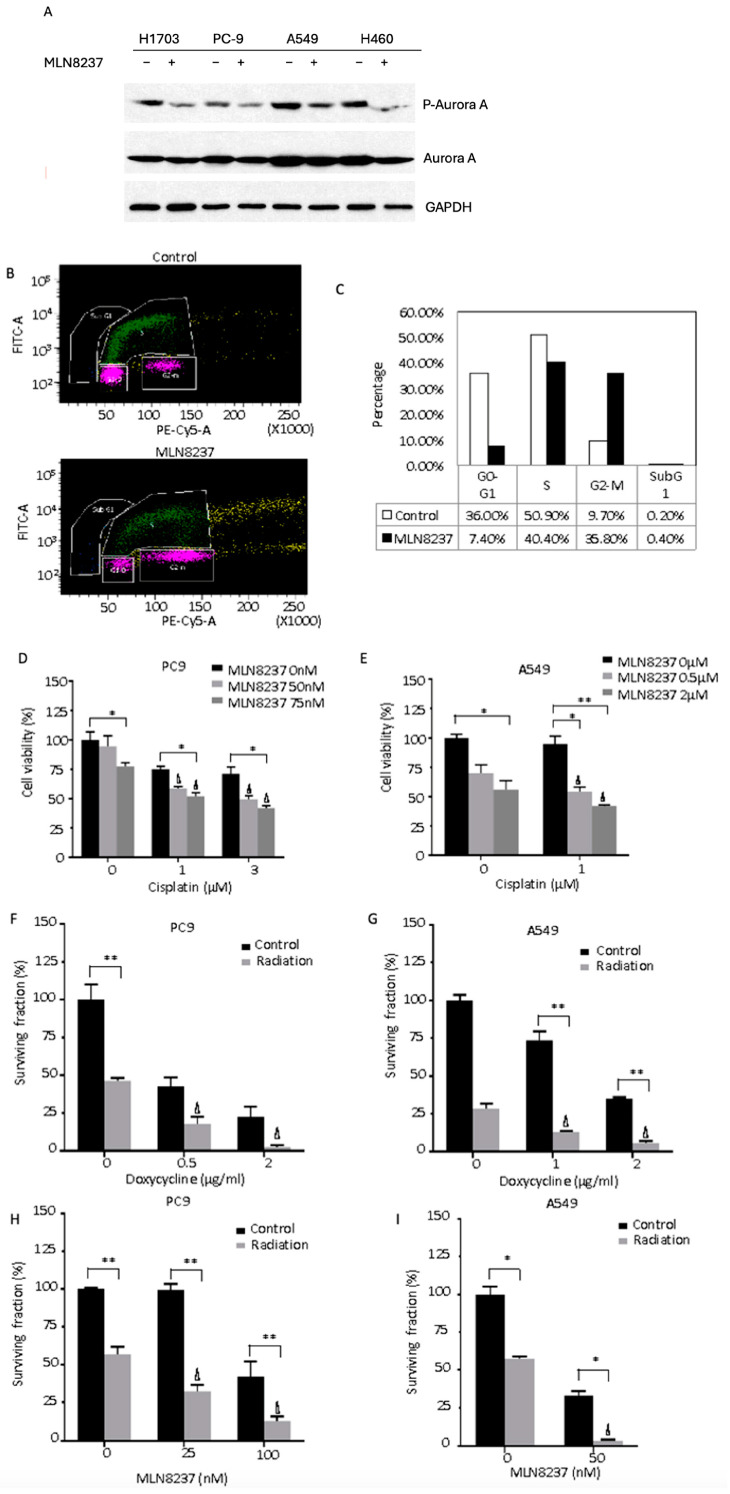
Inhibition of AURKA sensitizes lung cancer cells to cisplatin and radiation. (**A**) Western blotting: The expression of phospho-Aurora and total AURKA are shown with and without treatment of MLN8237 in H1703, PC9, A549, and H460 individually. (**B**) Flow cytometry demonstrating cell cycle stages of PC9 cells without treatment in the upper panel and with MLN8237 treatment in the lower panel. (**C**) The percentage of PC9 cells in G0-1, S, G2-M, sub-G1 cycle stage per flow cytometry with and without treatment of MLN8237. (**D**) The cell viability of PC9 cells after being treated with MLN8237 and/or cisplatin. (**E**) The cell viability of A549 cells after being treated with MLN8237 and/or cisplatin. (**F**) The surviving fraction of PC9 cells with and without treatment of doxycycline and/or radiation; (**G**) the surviving fraction of A549 cells with and without treatment of doxycycline and/or radiation; (**H**) the surviving fraction of PC9 cells with and without treatment of MLN8237 and/or radiation; (**I**) the surviving fraction of A549 cells with and without treatment of MLN8237 and/or radiation. The following notation was used to symbolize statistical significance in the figures: * *p* < 0.05, ** *p* < 0.01. Δ is used to denote a synergistic effect of combined treatments.

We examined the effects of MLN8237 on the cytotoxicity of cisplatin and radiation. To further confirm its activity in NSCLC cell lines, we analyzed the intensity of AURKA phosphorylation at the Thr288 site, which is located in the activation loop [5]. Our results showed that MLN8237 inhibits the phosphorylation of AURKA at the site of Thr288 in multiple NSCLC cell lines, such as PC9, A549, H1703, and H460 (Figure 3A). MLN8237 also led to cell cycle arrest in G2/M stage, resulting in an increased percentage of G2/M and a decreased percentage of G0-/G1-stage cells (Figure 3B,C). Similar to the AURKA knockdown experiments, MLN8237 increased the cytotoxicity of cisplatin synergistically (CI < 1) (Figure 3D,E).

AURKA knockdown (Figure 3F,G) and MLN8237 treatment (Figure 3H,I) worked synergistically with radiation in PC9 and A549 cells to increase cytotoxicity as demonstrated by colony formation assays (CI < 1). These data indicate that the inhibition of AURKA with cisplatin or radiation significantly reduces NSCLC cell survival in vitro.

### 3.3. AURKA Knockdown and MLN8237 Lead to an Increase in DNA Double-Strand Breaks and Apoptosis

**Figure 4:**
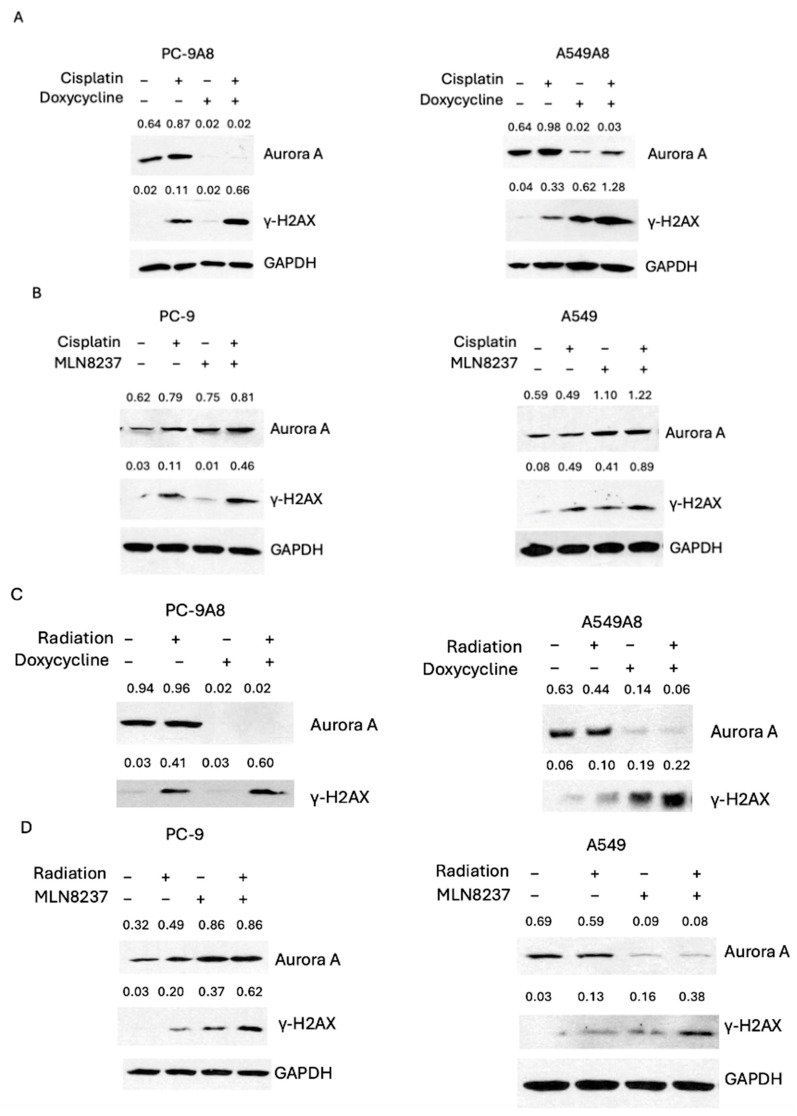
Blocking the function of AURKA increases cisplatin-induced DNA double-strand breaks. (**A**) Expression of AURKA, γ-H2AX, and GADPH in PC9A8 cells and A549A8 with and without cisplatin and doxycycline treatment by Western blot. (**B**) Expression of AURKA, γ-H2AX, and GADPH in PC9 cells and A549 with and without cisplatin and MLN8237 treatment by Western blot. (**C**) Expression of AURKA, γ-H2AX, and GADPH in PC9A8 and A549A8 cells with and without radiation and doxycycline treatment by Western blot. (**D**) Expression of AURKA, γ-H2AX, and GADPH in PC9 and A549 cells with and without radiation and MLN8237 treatment. The ratios of band intensities divided by GAPDH are noted at the top of each protein band.

We next explored whether AURKA knockdown could increase the DNA damage response induced by cisplatin or radiation treatment. Our results showed that γ-H2AX, used as a marker for DNA double-strand break, was increased in cell lines treated with the combination of AURKA knockdown and cisplatin or MLN8237 and cisplatin (Figure 4A,B). Similar results were also observed with the combination of AURKA knockdown and radiation, or MLN8237 and radiation (Figure 4C,D). The effect of AURKA knockdown and MLN8237 on the formation of γ-H2AX was similar. These data indicate that inhibiting the function of AURKA can sensitize NSCLC cells to cisplatin and radiation by modulating the DNA double-strand break response, which could eventually trigger cell death.

**Figure 5:**
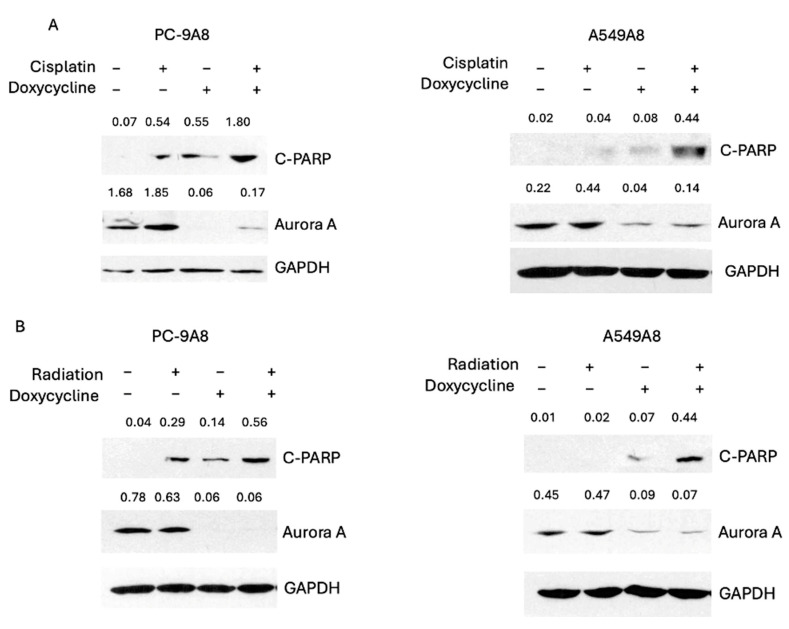
Knockdown of AURKA increases apoptosis induced by cisplatin or radiation. (**A**) Expressions of C-PARP, AURKA, and GADPH were shown in PC9 and A549 cells with and without the treatment of cisplatin and/or doxycycline for 48 h. (**B**) Expressions of C-PARP, AURKA, and GADPH were shown in PC9 and A549 cells (with and without the treatment of radiation and/or doxycycline for 48 h). The ratios of band intensities divided by GAPDH are noted at the top of each protein band.

We investigated apoptosis and senescence upon combination treatment to explore the underlying mechanism of cytotoxicity induced by inhibition of both AURKA and cisplatin. Our results indicate that apoptosis was detected, as marked by c-PARP, with a combination of AURKA knockdown and cisplatin or radiation (Figure 5A,B).

**Figure 6:**
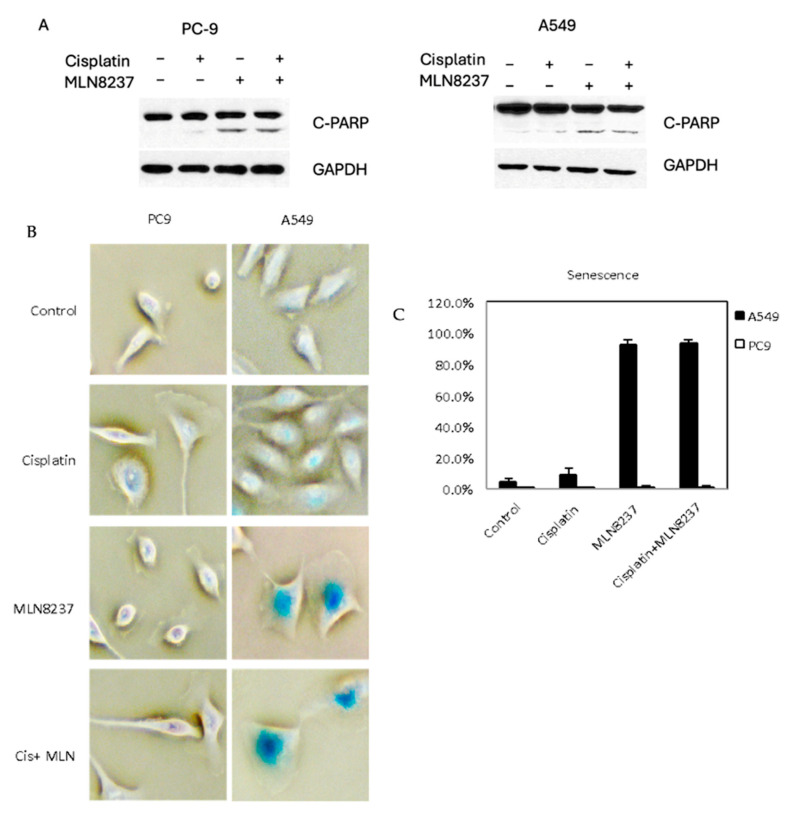
Inhibiting AURKA by MLN8237 induces apoptosis and senescence. (**A**) Western blotting: the expression of cleaved PARP and GADPH in PC9 and A549 cells with and without treatment of cisplatin and/or MLN8237 for 48 h. (**B**) Representative picture of staining of senescence-associated beta-galactosidase in PC9 and A549 cells with and without treatment of cisplatin and/or MLN8237. (**C**) Quantitative analysis of staining of senescence-associated beta-galactosidase in PC9 and A549 cells with and without treatment of cisplatin and/or MLN8237.

When treated with a combination of MLN8237 and cisplatin, apoptosis was more marked in the p53 mutant cell line, PC9, as opposed to a p53 wild-type cell line, A549 (Figure 6A). Upon treatment with MLN8237, the morphology of A549 cells changed significantly, becoming more flat and larger than untreated cells, which is a typical morphological change for senescence. Through the staining of senescence-associated beta-galactosidase, we found that significant senescence was induced in A549 cells but not in PC9 cells (Figure 6B,C).

### 3.4. Inhibition of AURKA Increases Expression of Selective Immune Checkpoints, Including PD-L1 and B7H3

**Figure 7:**
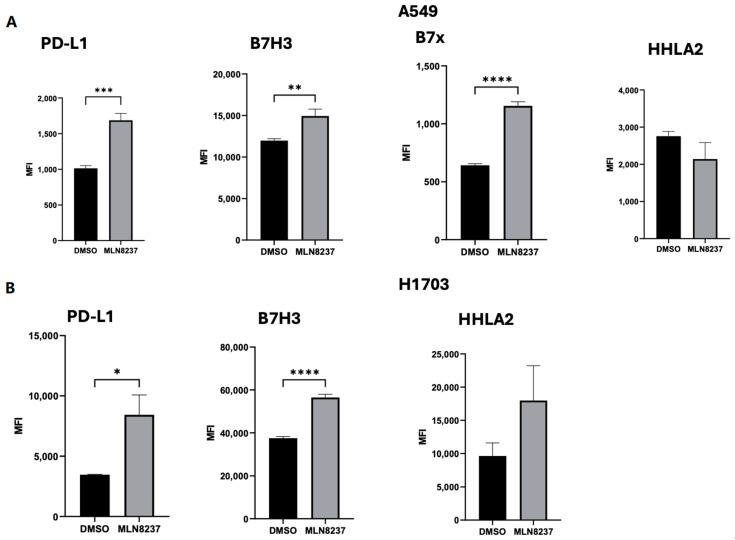
(**A**) Flow cytometric analysis of PD-L1, B7x, HHLA2, and B7H3 in the A549 cell line after treatment with 1 μM of MLN8237 for 72 h. (**B**) Flow cytometric analysis of PD-L1, HHLA2, and B7H3 in A549 cell line after treatment with 1 μM of MLN8237 for 72 h. The following notation was used to symbolize statistical significance in the figures: * *p* < 0.05, ** *p* < 0.01, *** *p* < 0.001 and **** *p* < 0.0001.

Recent studies have suggested that the suboptimal efficacy of AURKA inhibitors could be due to the compensatory activation of immune checkpoints [28]. Thus, we next investigated immune evasion mechanisms that could be triggered by MLN8237 by studying members of the group III molecules of the B7-CD28 immune checkpoint family. In order to explore the effect of MLN8237 on the expression of PD-L1, B7-H3, B7x, and HHLA2, we pursued flow cytometry analysis. Flow cytometry analysis revealed that inhibition of AURKA with MLN8237 led to a significant increase in PD-L1 and B7-H3 expression in both A549 and H1703 cell lines (Figure 7A,B). B7x expression was induced by MLN8237 treatment in A549 but was not detected at baseline in H1703.

## 4. Discussion

We previously conducted a genome-wide high-throughput RNAi screening to identify novel synergistic targets of cisplatin, whose modulation could affect sensitivity in lung cancer cells. Our prior study [4] identified YAP1 as a potential candidate to affect platinum sensitivity. In the current study, we investigate the role of AURKA in response to platinum and radiation treatment and its effect on the expression of immune checkpoints. Here, we demonstrate that knocking down AURKA in a doxycycline-inducible system or inhibiting AURKA by MLN8237 improves cisplatin and radiation activity in multiple lung cancer cell lines and in an in vivo xenograft model. This synergistic effect is likely secondary to an increase in DNA double-strand breaks and cell cycle arrest, leading to apoptosis and senescence. We also demonstrate that AURKA inhibition by MLN8237 leads to increased expression of immune checkpoints PD-L1 and B7-H3 in NSCLC cell lines.

Aurora kinase inhibition strategies are being heavily explored. Given the essential and various roles of Aurora kinases in cellular physiology, adverse events caused by toxicity in healthy cells have been difficult to manage. MLN8237 is the Aurora kinase inhibitor that has progressed the most in clinical evaluation so far and has reached phase III trials [38]. The efficacy of AURKA inhibitors as single agents in NSCLC clinical trials has been limited primarily to disease stabilization. A phase 1/2 study of single-agent MLN8237 in multiple heavily pretreated solid tumors showed an objective response in nine of forty-nine women with breast cancer, ten of forty-eight patients with small cell lung cancer, and one in twenty-three NSCLC patients. Seventeen of twenty-three NSCLC patients had stable disease in this trial. The adverse effects of single-agent alisertib were manageable; it caused grade 3-4 neutropenia in 43% and grade 3-4 anemia in 10% of patients [39].

Multitargeted Aurora kinase inhibitors may have benefits over highly selective agents since they may target potential resistance pathways, as well. However, this may come at a higher toxicity cost. For instance, AMG-900, a pan-AURK inhibitor, used in a phase I trial led to partial response in three out of twenty-nine patients with ovarian cancer. In this study, 75% of patients experienced grade 3 or higher adverse events, and 24% of patients discontinued AMG900 due to adverse events [40]. Most Aurora kinase inhibitors function by binding to the ATP binding site of kinases. Given the highly conserved nature of this site, increasing selectivity and specificity remains challenging [41]. Newer classes of AURK inhibitors like PROTACs are in development. These may address these concerns and inhibit the non-catalytic functions of Aurora kinases [42].

The marginal efficacy of the current single-agent inhibitors in clinical studies prompted the exploration of combination strategies. Given the suggested role of AURKA in the development of chemotherapy resistance, many combinations of Aurora kinase inhibitors with chemotherapy agents are being studied. A combination of MLN8237 with paclitaxel in second-line treatment of small-cell lung cancer (SCLC) in a phase II trial revealed a survival benefit in a subgroup with select genetic alterations. However, the grade 3-4 adverse events were seen in 67% of patients along with four reported treatment-related deaths [43]. Similarly, this combination was also studied in advanced breast cancer and recurrent ovarian cancer in a randomized phase III trial compared to paclitaxel alone for which the PFS favored the alisertib arm at the expense of increased toxicity [44].

Other combination strategies to enhance the AURKA inhibitors’ activity in NSCLC include adding targeted therapies. AURKA has been suggested as an adaptive resistance mechanism to the EGFR inhibitors [25]. Hence, osimertinib combined with MLN8237 in EGFR mutant NSCLC has been studied in clinical trials, reaching a median PFS of 5.5 months [45]. AURKA has been implicated in the tumorigenesis of KRAS mutant NSCLC as well [46], which encouraged the combinatory use of the AURKA inhibitor, VIC-1911, with sotorasib for KRAS G12C mutant NSCLC [47].

Prior studies of MLN8237 in NSCLC cell lines have demonstrated that the inhibition of AURKA activation decreased cell viability and senescence, as determined by cell morphological changes [22]. Another study suggested that the combination of MLN8237 and cisplatin in cisplatin-resistant NSCLC cell lines led to decreased cell viability and increased apoptosis, as determined by Annexin V staining [23]. Our study expands on the potential combinatory strategies of AURKA inhibition and explores underlying cellular mechanisms. It reveals synergistic cytotoxic activity with inhibition of AURKA and cisplatin or radiation in NSCLC. In order to understand the underlying mechanisms leading to enhanced cytotoxicity with AURKA inhibition, we studied DNA double-strand break formation, cell cycle progression, apoptosis, and senescence pathways. We demonstrate that the use of MLN8237 led to a block of cell cycle progression at the G2/M phase. Prior studies have suggested that the mechanism of cisplatin and radiation resistance conferred by AURKA depends partly on the DNA double-strand break repair system. Overexpression of AURKA has been shown to decrease DNA repair molecules such as BRCA1/2 and γH2AX and lead to a decrease in apoptosis [48]. Here, in our study, we demonstrate that treatment with AURKA inhibitor MLN8237 or AURKA knockdown leads to an increase in DNA double-strand breaks in NSCLC cell lines, which becomes more pronounced when combined with cisplatin or radiation therapy.

Our study also demonstrates that the cell cycle arrest and accumulated DNA double-strand breaks ultimately lead to apoptosis and/or senescence, possibly dependent on the TP53 mutation status of the cells. The interplay between p53 and AURKA has indeed been suggested in multiple prior studies. Phosphorylation of p53 by AURKA has been reported to lead to the activation or inhibition of p53 by affecting its interaction with MDM2, depending on the phosphorylation site [49,50]. Our study here also suggests that TP53’s mutational status may determine the cell’s fate upon treatment with MLN8237, whether it leads to senescence or apoptosis.

More recently, studies proposed that the suboptimal efficacy of AURKA inhibitors may be related to the compensatory activation of immune checkpoints. This concept is especially relevant in NSCLC, given the frequent use of immunotherapy in treatment. Our study demonstrates an increase in PD-L1 and B7-H3 expression in NSCLC cell lines upon treatment with MLN8237. These pathways may act as immune evasion mechanisms that decrease the efficacy of AURKA inhibition. Future studies should explore the expression of these immune checkpoints in in vivo models treated with AURKA inhibitors. The downstream effects of these immune checkpoint changes on the tumor immune microenvironment should be investigated in in vivo models. There are multiple clinical trials investigating the efficacy and safety of B7-H3 targeting strategies in various malignancies [51]. The use of MLN8237 in combination with immunotherapy treatments targeting the PD-1/PD-L1 pathway or B7-H3 pathway should be investigated in in vivo lung cancer models since these combinations may lead to synergistic and more durable anti-tumor effects. Clinical trials using AURKA inhibitors should incorporate translational studies that investigate activated immune evasion pathways in post-treatment samples. These studies can better guide combination strategies using AURKA inhibition and immune checkpoint inhibitors.

## 5. Conclusions

In summary, this study provides findings to support AURKA as a key modulator of sensitivities to platinum and radiation. We demonstrate that inhibiting AURKA could sensitize NSCLC to cisplatin and radiation. In addition, we show that AURKA inhibition stimulates the induction of multiple actionable immune checkpoint pathways. These findings should be further investigated. These findings may aid in rationally designed combination therapies in the NSCLC treatment landscape, which has been significantly transformed by the adoption of immunotherapy.

## Figures and Tables

**Table 1:** Distribution of AURKA staining based on histology and mutation status.

	Negative *n* (%)	Weak Positive *n* (%)	Moderate–Strong Positive *n* (%)	Total Positive *n* (%)
All cases (*n* = 94)	8 (8.5%)	54 (57.5%)	32 (34.0%)	86 (91.5%)
Histology				
Adenocarcinoma (*n* = 55)	5 (9.1%)	33 (60.0%)	17 (30.9%)	50 (90.9%)
Squamous cell carcinoma (*n* = 15)	0 (0%)	9 (60.0%)	6 (40.0%)	15 (100%)
Large-cell carcinoma (*n* = 16)	2 (12.5%)	7 (43.8%)	7 (43.8%)	14 (87.5%)
Other (*n* = 8)	1 (12.5%)	5 (62.5%)	2 (25.0%)	7 (87.5%)
Mutation status				
EGFR or KRAS mutated (*n* = 23)	1 (4.3%)	17 (73.9%)	5 (21.7%)	22 (95.6%)
EGFR/KRAS wild type (*n* = 30)	2 (6.7%)	18 (60.0%)	15 (33.3%)	33 (93.3%)

## Data Availability

The supporting data are not publicly available due to research participant privacy restrictions.

## Supplementary Materials

The following supporting information can be downloaded at: https://www.mdpi.com/article/10.3390/cancers16162805/s1, Figure S1: MTS and Colony Formation assays for A549A8.
