# Supplementary figures and images for: Aurora Kinase A Inhibition Potentiates Platinum and Radiation Cytotoxicity in Non-Small-Cell Lung Cancer Cells and Induces Expression of Alternative Immune Checkpoints
Source: Cancers (Basel). 2024 Aug 9;16(16):2805. doi: 10.3390/cancers16162805 (PMC11352996; doi:10.3390/cancers16162805)

Supplementary Figure S1. MTS and Colony Formation assays for A549A8

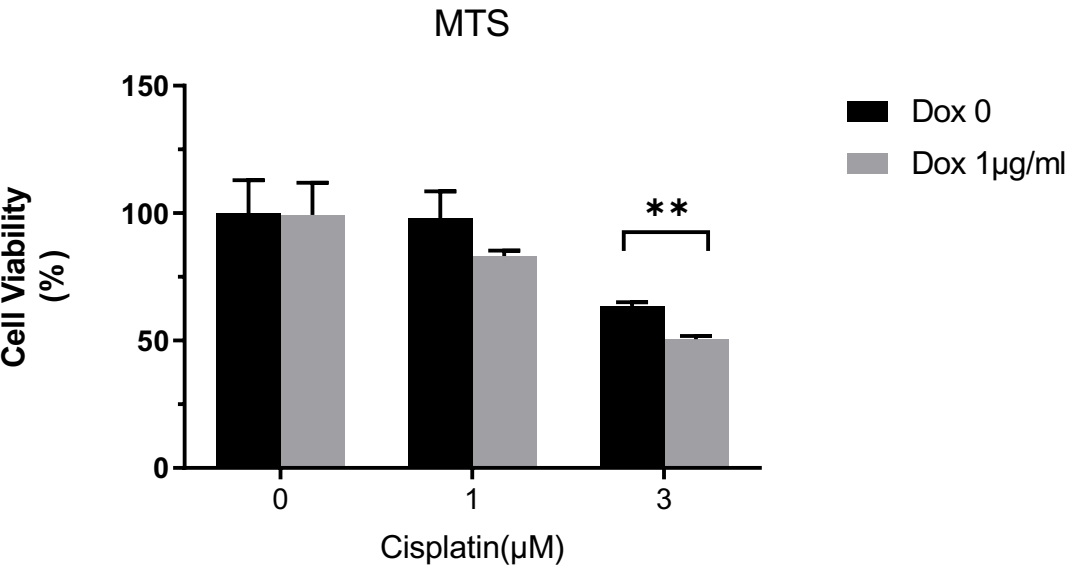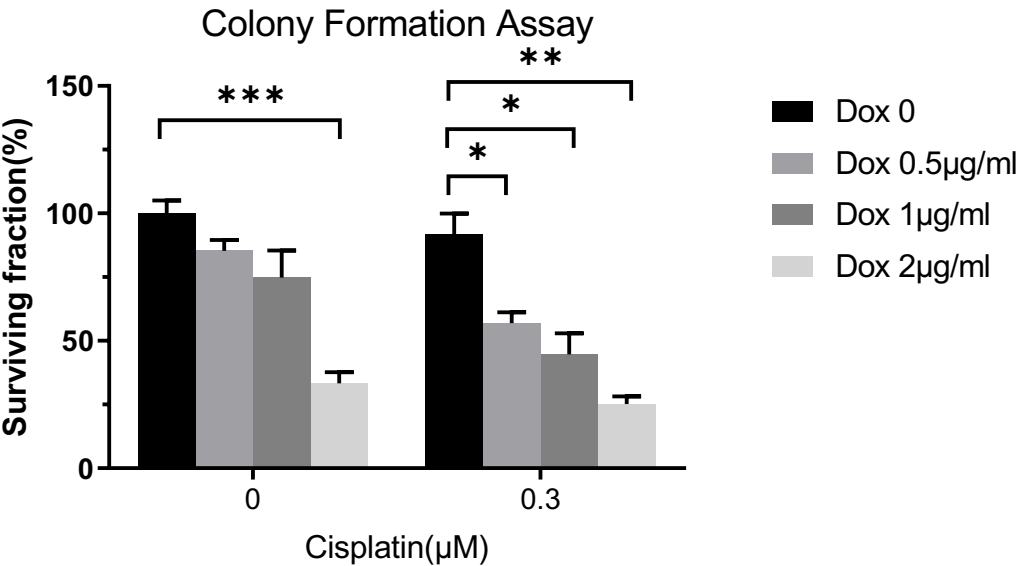

Supplement: Supplementary file 1 [file cancers-16-02805-s001.zip › cancers-3097760-supplementary.pdf]
